# Close coupling of plant functional types with soil microbial community composition drives soil carbon and nutrient cycling in tundra heath

**DOI:** 10.1007/s11104-023-05993-w

**Published:** 2023-03-27

**Authors:** Marianne Koranda, Riikka Rinnan, Anders Michelsen

**Affiliations:** 1 Division of Terrestrial Ecosystem Research, Centre for Microbiology and Environmental Systems Science, University of Vienna, Djerassiplatz 1, 1030 Vienna, Austria; 2 Terrestrial Ecology Section, Department of Biology, University of Copenhagen, 2100 Copenhagen, Denmark; 3 Center for Permafrost (CENPERM), University of Copenhagen, 1350 Copenhagen, Denmark

**Keywords:** Carbon and nutrient cycling, Microbial community, Moss, Plant-soil-microbe interactions, Shrub, Tundra

## Abstract

**Aims:**

This study aimed at elucidating divergent effects of two dominant plant functional types (PFTs) in tundra heath, dwarf shrubs and mosses, on soil microbial processes and soil carbon (C) and nutrient availability, and thereby to enhance our understanding of the complex interactions between PFTs, soil microbes and soil functioning.

**Methods:**

Samples of organic soil were collected under three dwarf shrub species (of distinct mycorrhizal association and life form) and three moss species in early and late growing season. We analysed soil C and nutrient pools, extracellular enzyme activities and phospholipid fatty acid profiles, together with a range of plant traits, soil and abiotic site characteristics.

**Results:**

Shrub soils were characterised by high microbial biomass C and phosphorus and phosphatase activity, which was linked with a fungal-dominated microbial community, while moss soils were characterised by high soil nitrogen availability, peptidase and peroxidase activity associated with a bacterial-dominated microbial community. The variation in soil microbial community structure was explained by mycorrhizal association, root morphology, litter and soil organic matter quality and soil pH-value. Furthermore, we found that the seasonal variation in microbial biomass and enzyme activities over the growing season, likely driven by plant belowground C allocation, was most pronounced under the tallest shrub *Betula nana*.

**Conclusion:**

Our study demonstrates a close coupling of PFTs with soil microbial communities, microbial decomposition processes and soil nutrient availability in tundra heath, which suggests potential strong impacts of global change-induced shifts in plant community composition on carbon and nutrient cycling in high-latitude ecosystems.

**Supplementary Information:**

The online version contains supplementary material available at 10.1007/s11104-023-05993-w.

## Introduction

Plant communities in Arctic ecosystems are characterized by a high diversity of growth forms or ‘plant functional types’ (sensu Chapin et al. 1996), comprising evergreen and deciduous shrubs, graminoids, forbs and mosses, which differ in their response to environmental factors and their influence on ecosystem functioning (Dorrepaal 2007). Observations during the last 20 years have shown that the composition of plant communities in high-latitude ecosystems is shifting due to global change (Elmendorf et al. 2012; Myers-Smith et al. 2020): The abundance of deciduous shrubs is increasing (‘arctic greening’), due to longer growing seasons and enhanced soil nutrient availability (Myers-Smith et al. 2011; Mekonnen et al. 2021) (but see also Vowles and Björk (2019) for expansion of evergreen shrubs), while moss abundance tends to decline (Lang et al. 2012; Sorensen et al. 2012), partly caused by increased shading by vascular plants (Van der Wal et al. 2005; Jägerbrand et al. 2012). Such shifts in plant community composition may exacerbate or alleviate effects of climate change on ecosystem functioning, thus potentially surpassing direct effects of global warming on carbon and nutrient cycling and ecosystem carbon storage (Wookey et al. 2009). For example, the spread of deciduous shrubs may enhance decomposition of soil organic matter (SOM) due to increased belowground C allocation (‘priming’) (Street et al. 2020; Parker et al. 2021), but also promote ecosystem C storage by production of lignin-rich recalcitrant litter (Mekonnen et al. 2018). Increased shrub abundance in tundra ecosystems may influence soil temperature in winter (‘snow-shrub-hypothesis’) (Sturm et al. 2005; Way and Lapalme 2021) and in summer via effects on albedo and surface energy budget (Blok et al. 2010; Kropp et al. 2021), with important implications for soil microbial activity and nutrient availability and hence feedback on ecosystem carbon cycling.

**Fig. 1:**
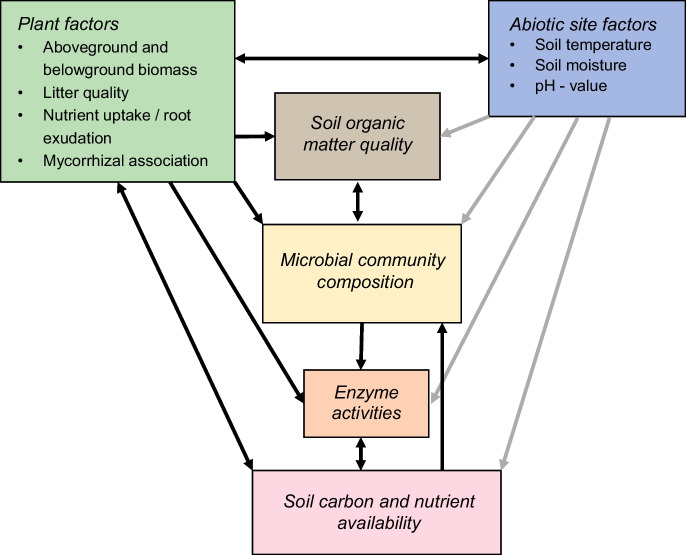
Simplified overview of the interactions between plant traits, soil microbial communities and soil functions (not depicted are interactions with the soil faunal community). Arrows in black indicate relationships highlighted in this study. Plant factors include (1) functional traits related to *aboveground and belowground plant biomass* (e.g., specific leaf area, relative growth rate, the ratio of aboveground to belowground biomass and depth and morphology of the root system), (2) characteristics of *litter quality* (e.g., litter C:N ratio, lignin content and concentration of plant secondary metabolites like tannins), (3) *plant nutrient uptake* (i.e., magnitude and timing of nutrient uptake and preference for the chemical form of nutrients) and the quantity and chemical quality of *root exudates,* (4) *mycorrhizal association* of the plants (including nutrient foraging capacity of the fungal symbiont and morphological characteristics of the mycelium). Site factors are on the one hand abiotic factors, but are also strongly influenced by plant traits: Plants affect (1) the *soil thermal regime* via shading, influence on albedo, the insulation capacity of plant biomass and via snow trapping in winter, (2) *soil moisture* via influence on evapotranspiration and root uptake of water, (3) *soil pH-value* via acidifying effects of plant nutrient uptake and litter decomposition. Soil organic matter quality is determined by the quantity and quality of plant litter input, and by the soil microbial community via microbial necromass formation. The structure of the soil microbial community depends on the nature of available complex substrates, i.e., litter and SOM quality, on the availability of labile C and nutrients, and on abiotic site factors like soil pH. Microbial community composition can also be influenced by plant secondary metabolites via antagonistic or beneficial effects. The production of extracellular enzymes by soil microbes reflects the microbial community structure, but is also regulated by soil microbes depending on substrate supply and nutrient availability. Enzyme activities are also affected by abiotic factors like soil temperature and moisture, and plants may contribute to the soil enzyme pool via production of phosphatases. The concentration of easily available carbon and nutrients in soil results from the balance of enzyme activities, microbial uptake and release of C and nutrients, and plant nutrient uptake and C exudation

The impact of plant species and plant functional types on soil microbial activity and C and nutrient cycling is determined by complex interactions between plant characteristics or ‘plant functional traits’, abiotic site factors, SOM quality and soil microbial communities (Fig. 1). Despite considerable research interest during the last decades (e.g., De Deyn et al. 2008; Legay et al. 2014; Fry et al. 2019; Weil et al. 2021), these complex interactions between plant traits and soil functions are still not fully understood.

In this study we focused on two plant functional types (in a broad sense) which are dominant in many (sub-)arctic ecosystems, namely dwarf shrubs and mosses. Mosses have been almost neglected in studies of plant-soil interactions, although bryophytes are distinct from vascular plants in various respects, including their impact on ecosystem functioning (Turetsky 2003). Bryophytes strongly impact soil microbial activity and soil nutrient availability by influencing soil temperature and moisture (Soudzilovskaia et al. 2013; Koranda and Michelsen 2021), sequestering atmospheric nitrogen deposition (Gundale et al. 2011; Koranda and Michelsen 2021) and via association with N-fixing symbionts (Lindo et al. 2013; Rousk et al. 2013). Furthermore, mosses produce slowly-decomposing, recalcitrant litter (Hobbie 1996; Lang et al. 2009), but also provide labile substrates for soil microbes via leaching of intracellular metabolites (Slate et al. 2019). In this study we selected three moss species differing in morphology and microsite preference and compared their influence on soil functioning with three dwarf shrub species of distinct life form (evergreen versus deciduous) and mycorrhizal association. We aimed at elucidating (1) if plant functional types and plant species differ in their effects on soil microbial communities, extracellular enzyme activities and soil nutrient availability, and (2) which (plant- and abiotic) factors are responsible for these differences in soil functioning. For this purpose, we took soil samples under three widespread moss species (*Hylocomium splendens, Aulacomnium turgidum* and *Tomentypnum nitens*) and three shrub species (*Empetrum hermaphroditum, Arctostaphylos alpinus* and *Betula nana*) in a tundra heath in Northern Sweden in early and late growing season. We analysed soil samples for microbial community composition, extracellular enzyme activities and labile C and nutrient pools and also determined a wide range of plant characteristics (including plant biomass, litter quality), SOM quality and soil physicochemical factors. While such an observational approach does not allow to clearly distinguish direct effects of plant species from abiotic effects related to site preference of plant species, it enabled us to study the long-term influence of plant species and plant functional traits on the SOM quality and soil microbial community, which would not be possible in a manipulative study, given the very slow plant growth, litter decomposition and soil formation in the Arctic. We hypothesized (1) that microbial decomposition activity and soil nutrient availability would be lower under mosses compared to shrubs, due to the poor litter quality of mosses and antimicrobial effects of secondary metabolites in moss biomass; (2) that soil under *Betula nana* would exhibit higher microbial biomass and extracellular enzyme activities compared to ericaceous dwarf shrubs, because of high photosynthetic leaf area and thus high belowground C allocation of *B. nana* and high content of phenolics in ericaceous shrub litter.

## Materials and methods

### Study site

**Fig. 2:**
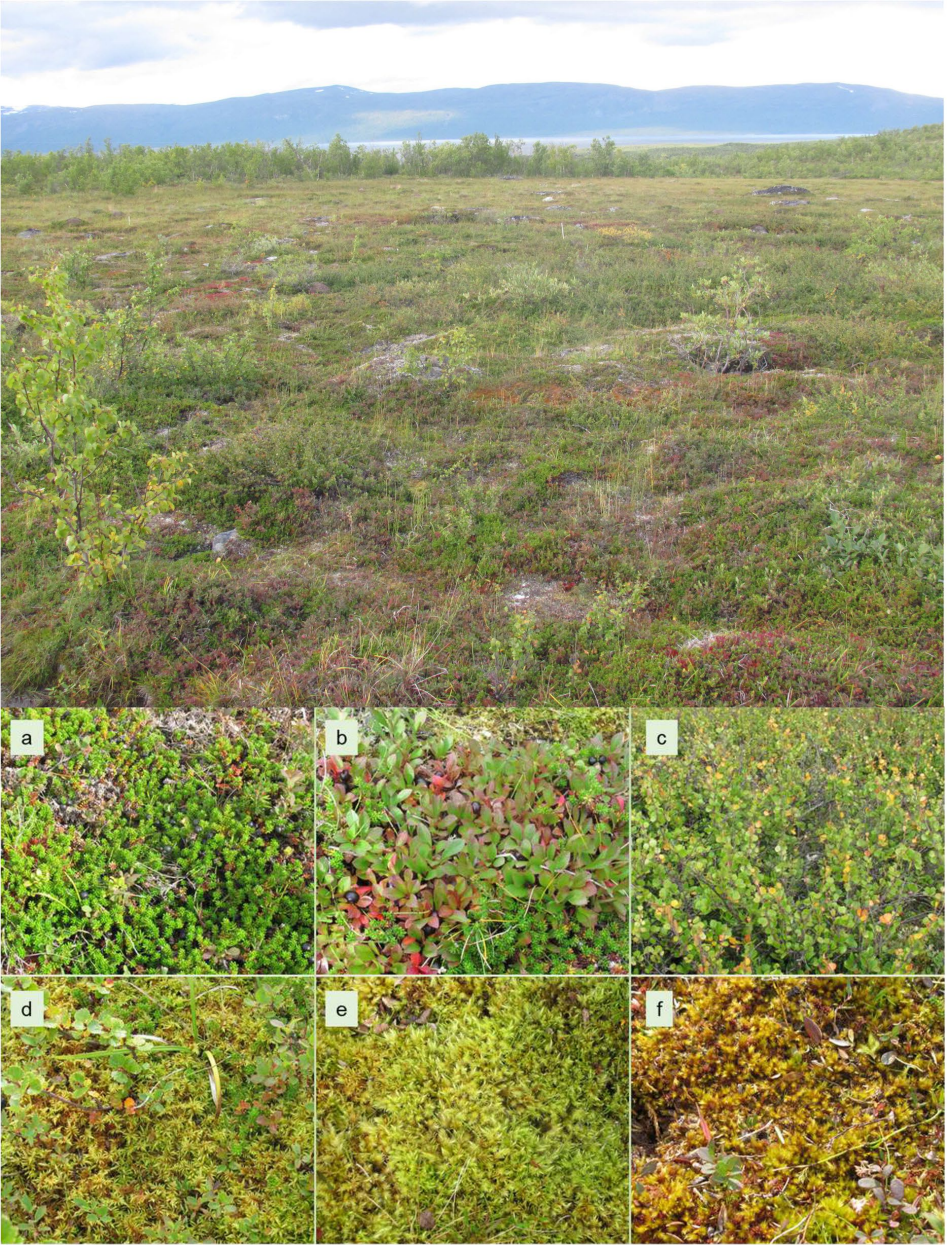
Tundra heath study site in Northern Sweden. Dwarf shrub and moss species selected for the study: **a** *Empetrum hermaphroditum*, **b** *Arctostaphylos alpinus*, **c** *Betula nana*, **d** *Hylocomium splendens*, **e** *Aulacomnium turgidum*, **f** *Tomentypnum nitens*

The study was performed in a tundra heath located close to Abisko in northern Sweden (68°20′24.7’’ N, 18°50′35.5’’ E). We chose a study site characterized as dwarf shrub tundra (Fig. 2) located below the tree line and surrounded by open mountain birch forest (*Betula pubescens* var. *pumila* L.). Five replicate blocks (size between 10 × 10 m and 20 × 20 m) with similar vegetation composition were selected within an area of 200 × 200 m. Vegetation at the study site was dominated by evergreen and deciduous dwarf shrubs (*Empetrum hermaphroditum, Betula nana, Arctostaphylos alpinus, Vaccinium uliginosum*), mosses (*Hylocomium splendens, Tomentypnum nitens, Aulacomnium turgidum* and *Sphagnum* spp.), and scattered grasses, sedges and forbs. The vegetation structure was characterized by a patchy distribution of plant species. In contrast to other tundra types (e.g., tussock tundra), the distribution of plant species at the study site was not strictly related to microtopography, although some microsite preferences of plant species were apparent (i.e., troughs and wetter sites were preferentially grown by mosses, and hummocks dominated by ericaceous shrubs). Soil type was classified as histosol, consisting of an organic horizon of 8 – 12 cm depth underlain by glacial till. Mineral horizon was mostly absent or very shallow. Bedrock in the Abisko region consists of mica schists with dolomite outcrops. Yearly precipitation for 2017—2018 was 340 mm and average air temperature was 13.1 °C in July and -11.0 °C in January (climate data from Abisko Research Station).

### Soil samplings

Soil samplings were performed on August 29^th^ and 30^th^ 2017 (late growing season) and on July 3^rd^ and 4^th^ 2018 (early growing season). Leaves of the deciduous plant species were already fully developed in late June. The time point end of August corresponded with the start of leaf senescence (or right before the start of senescence, depending on the plant species). At each time point soil cores (4 cm diameter) of the entire organic horizon (8 – 12 cm depth) were taken under three dwarf shrub species and three moss species (Table 1, Fig. 2a-f). Sampling sites were either monospecific with respect to the occurring plant species or dominated by one plant species. In each replicate block three subsamples (soil cores) per plant species were taken and bulked. Soil cores of the second sampling campaign were taken close to those of the first sampling (ca. 10 cm distance), in order to avoid spatial variability blurring seasonal differences. Regarding soil cores from moss grown sites, we defined soil beginning from the zone of partly decomposed moss, which was usually separated from the top layer of undecomposed brown moss by a clearly identifiable border. Soil cores from moss sites typically exhibited a gradient of increasing degree of decomposition and increasing darkness in brown to black colour downwards in the organic horizon.

**Table 1:** Characteristics of dwarf shrub and moss species selected for the study

Plant species	Growth form / morphology	Mycorrhizal type
Dwarf shrubs		
*Empetrum hermaphroditum* (Hagerup)	Evergreen shrub / prostrate	Ericoid mycorrhiza
*Arctostaphylos alpinus* (Spreng.)	Deciduous shrub / prostrate	Arbutoid mycorrhiza ^1^
*Betula nana* (L.)	Deciduous shrub / erect	Ectomycorrhiza
Mosses		
*Hylocomium splendens* (Schimper)	Feathermoss / pleurocarpous	-
*Aulacomnium turgidum* (Schwaegr.)	Dense cushions / acrocarpous	-
*Tomentypnum nitens* (Loeske)	Dense cushions / pleurocarpous	-

^1^ Arbutoid mycorrhiza shares characteristics of ericoid and ectomycorrhiza and has only been described for the genera Arctostaphylos, Arbutus and Pyrola (Smith and Read 2008)

After soil sampling, roots were removed, soil was homogenized by hand and stored at 4 °C until further analyses. Soil extractions were performed within two days and enzyme assays within seven days after soil samplings.

### Soil physicochemical factors

Soil temperature was measured manually using thermometers at 5 cm soil depth at four time points between early July and end of August. At each time point three measurements per block and plant species (corresponding with sampling subsites) were performed and averaged (i.e., 3 × 5 measurements per time point and plant species). Measurements were done in the afternoon; hence values represent approximate daily maximum soil temperatures. Soil moisture was determined gravimetrically at the soil samplings. Soil pH-value was determined in soil slurries (3 g fresh soil in 25 mL water).

### Soil characteristics

Subsamples of soil were freeze-dried, ground in a ball mill and analysed for total soil C and N by an Eurovector elemental-analyzer.

Soil organic matter quality was assessed from Fourier transform near-infrared (FT-NIR) spectra. Freeze-dried, ground soil was filled into glass vials, and samples were analysed by an Antaris II FT-NIR Analyzer (Thermo), with a resolution of 16 wavelengths cm^−1^ and 32 scans per sample. NIR spectra were subjected to Standard Normal Variate (SNV) pre-processing to remove undesired scatter effects, centered and subjected to principal component analysis (PCA) using SIMCA 16. We assessed the loadings in order to assign the main spectral features to likely chemical components according to Workman and Weyer (2012). As the first PCA axis mainly represented differences in water content of ground samples resulting from sample storage, we only report the sample scores of the second PCA axis, which represented the greatest proportion of the variability in SOM quality.

The concentration of condensed tannins in soil was determined by the acid-butanol method, modified after Booker et al. (1996) and Smolander et al. (2005). Briefly, freeze-dried, ground soil was extracted with 70% acetone (containing 0.1% ascorbic acid). Extracts were evaporated to dryness and re-dissolved in water. Aliquots were mixed with acid butanol (5% HCl in 1-butanol, v/v), incubated at 95 °C for 1.5 h, and absorbance at 550 nm was measured with a spectrophotometer. Standards were prepared using commercially available procyanidin B2 (Sigma) diluted in water and processed like soil extracts.

### C and nutrient pools

Subsamples of fresh soil were extracted with 0.5 M K_2_SO_4_ (1:10, w/w) and filtered through ash free paper filters (Whatman nr. 42). Concentrations of dissolved organic C and total dissolved N were measured with a TOC/TN analyzer (Shimadzu). Dissolved organic N concentration was calculated from the difference of total dissolved N and inorganic N.

Concentrations of NH_4_^+^, NO_3_^−^ and PO_4_^−^ were determined by flow-injection analysis (Fiastar 5000, FOSS analytical, Höganäs, Sweden), using applications AN 5220 for NH_4_^+^, AN5201 for NO_3_^−^ and AN5240 for PO_4_^−^, respectively. Three data points were excluded (one for NH_4_^+^, two for PO_4_^−^) because of problems with analysis resulting from precipitation in the extracts.

Microbial biomass was determined by the fumigation-extraction method (Brookes et al. 1985). Microbial biomass C, N and P was calculated from the difference in concentrations of dissolved organic C, total dissolved N and PO_4_^−^, respectively, in extracts of fumigated and non-fumigated soil samples. An extraction coefficient of 0.45 for C (Wu et al. 1990) and 0.4 for N and P (Jonasson et al. 1996) was used to account for incomplete extraction of microbial biomass C and N.

### Microbial community structure

The abundance of microbial groups was estimated from phospholipid fatty acids (PLFAs) using a modified method after Buyer and Sasser (2012). After extraction of freeze-dried soil samples by a mixture of methanol, chloroform and citrate buffer (2:1:0.8, v/v/v), PLFAs were separated from neutral lipids on silica columns and subjected to alkaline methanolysis. Dried fatty acid methyl esters were re-dissolved in isooctane and concentrations of PLFAs were determined on a gas chromatograph (Trace GC Ultra, Thermo Scientific) equipped with a DB-23 column. A mixture of fatty acid methyl esters (FAMEs) (Supelco, nr. 47,080-U and 47,885-U) was used as a qualitative standard. An internal standard (19:0) was used for calculation of FAME concentrations. We used the PLFAs i15:0, a15:0, i16:0, i17:0, a17:0 as indicators of Gram + bacteria (Firmicutes), 10Me17:0 as indicator of Actinobacteria and 16:1ω9, 16:1ω7, cy17:0, cy19:0, 18:1ω7 as indicators of Gram- bacteria. PLFA 18:1ω9trans was used as (anaerobic) bacterial marker, as this fatty acid has recently been determined in high concentrations in a taxon of Deltaproteobacteria (S. Gorka, unpublished data), but has also been mentioned as marker for Clostridia (Firmicutes) (Ferguson et al. 2016). PLFA 18:2ω6,9 was used as indicator of ecto- and ericoid mycorrhizal and saprotrophic fungi (Frostegård et al. 2011; De Vries et al. 2012). We calculated a relative measure of fungi-to-bacteria ratio from the abundance of PLFA 18:2ω6,9 and the sum of bacterial indicator PLFAs (Bardgett et al. 1996). PLFAs 18:1ω9cis and 18:3ω3,6,9 were classified as general fungal markers, and 16:1ω5 as marker for arbuscular mycorrhizal fungi.

### Extracellular enzyme activities

Potential hydrolytic enzyme activities were estimated by microplate assays using fluorescent substrates, as described in detail in Koranda and Michelsen (2021). Soil slurries were prepared using Na-acetate buffer (pH 5.7). We chose an intermediate buffer pH-value (respective to the measured pH-values at the study sites) in order to ensure substrate stability in the assays (Niemi and Vepsäläinen 2005). It should be noted that these enzyme assays aim at measuring maximum enzyme activities at saturating substrate concentrations and under standard conditions (as a measure for the amount of enzymes present in soil), and not in-situ enzyme activities. The substrates 4-MUF-β-D-glucopyranoside, 4-MUF-β-D-cellobioside, 4-MUF-N-acetyl-β-D-glucosaminide, 4-MUF-phosphate and L-leucine-7-amino-4-methyl-coumarin were added for determination of β-glucosidase, cellobiosidase, chitinase, phosphatase and peptidase activities, respectively. Standard curves were prepared from 4-methylumbelliferone (MUF) and 7-amino-4-methylcoumarin in three concentrations. Microplates were incubated at 10 °C for 4 – 5 h (depending on the substrate), then fluorescence was measured at 365 nm excitation and 450 nm emission.

Potential oxidative enzyme activities (phenoloxidase and peroxidase) were measured photometrically using dihydroxyphenylalanine (DOPA) as substrate, as described previously (Koranda and Michelsen 2021).

### Plant biomass (including litter)

Aboveground plant biomass was determined in late July 2019, i.e. one year after the second soil sampling. The difference in time points is unlikely to have influenced our findings, as growth and decomposition rates of shrubs and mosses in tundra are very low. Total aboveground plant biomass was destructively harvested in four replicate plots of 50 × 50 cm size (*B. nana*), 25 × 25 cm (ericaceous shrubs) and 20 × 20 cm (mosses), respectively, dried at 60 °C in a drying oven and weighed. We did not separate green and brown moss, thus values comprise both living moss and moss litter. We therefore also included shrub litter in the biomass calculations of shrub plots, which, however, only accounted for a small proportion of aboveground shrub biomass (M. Koranda, pers. obs.).

Root biomass was determined from soil cores in early July 2018. Roots with diameter < 1 mm were considered as fine roots, thicker roots as coarse roots. As very thick roots were generally avoided when taking the soil cores, coarse root biomass of *B. nana* may be underestimated. Roots were washed, dried at 60 °C and weighed.

### Leaf and leaf litter characteristics

Leaf litter was collected in late September 2017. We collected senescent leaves of dwarf shrubs which were still attached to the plants, and samples of brown moss (three replicate samples per plant species). While leaf litter accounts for only a minor proportion of total vascular plant litter mass, it generally exhibits higher variation in chemical quality among plant species compared to root litter (Hobbie 1996; McLaren et al. 2017). Samples of green leaves and green moss (three replicates) were collected in mid-growing season (end of July). All leaf and litter samples were dried at 60 °C and ground in a ball mill.

Total C and N in leaf and litter samples were determined by an elemental-analyzer (Eurovector). A characterization of chemical quality of litter samples was obtained using Fourier transform near-infrared (FT-NIR) spectroscopy, as described above for soil samples. Concentrations of condensed tannins in leaf litter were determined using the protocol described above for soil samples.

### Data analyses

Data were checked for normality and homogeneity of variance prior to analyses and square-root or log-transformed, if necessary. We assessed effects of plant species on plant biomass, leaf and leaf litter characteristics, soil characteristics and abiotic site factors by mixed-effect model ANOVA, with plant species as fixed factor and block as random factor, followed by Tukey’s post-hoc tests. Soil C and nutrient pools, abundance of PLFAs and extracellular enzyme activities were analysed by mixed-effect model ANOVA with plant species and season as fixed factors, and block and sampling subsite nested within block as random factors. Additionally, we also ran models with plant functional type (here in the sense of shrubs versus moss) and season as fixed factors, and plant species, block and sampling subsite nested within block as random effects. Differences between plant species in 2-way ANOVA models were assessed by Tukey’s post-hoc test. We calculated the explained variance of mixed-effect models using marginal R^2^ (fixed effects only) and conditional R^2^ (fixed and random effects) (Nakagawa and Schielzeth 2013).

We applied multivariate ordination techniques for estimating effects of plant species and season on soil microbial community composition, soil C and nutrient pools and enzyme activities. We used principal component analysis (PCA) for analysis of relative abundances of 18 PLFAs (mol %, square-root transformed), and for analysis of C and nutrient pools and enzyme activities (square-root transformed and standardized data). Effects of plant species and season on microbial community composition, C and nutrient pools and enzymes were further estimated by multivariate analysis of variance (PERMANOVA) of distance matrices based on Euclidian distances. The relationships between microbial community structure and the pattern of C and nutrient pools and enzyme activities were assessed using Mantel-tests of distance matrices.

In order to elucidate which plant-, soil- and site-factors explained the variation in soil microbial community composition under the six plant species, we performed mixed model regression analysis, with the ratio of fungal-to-bacterial PLFAs and the PC1 scores of the PLFA ordination, respectively, as dependent variables, and plant biomass and litter characteristics, soil characteristics and abiotic site factors, respectively, as explanatory variables. As the explanatory variables were either determined only once per season (plant traits) or showed little seasonal variation (soil characteristics and abiotic site factors), microbial community data from early and late season sampling were averaged and regression analyses performed with summer season averages of all data. Linear mixed-effect models were then run separately with plant factors, soil factors and site factors, respectively, as fixed effects and plant species as random effect. We selected the final models using stepwise backward selection by removing non-significant explanatory factors and based on the Akaike-information criterion (AIC) of the models.

All statistical analyses were performed using R version 3.5.1 (R Core Team 2018), with the packages ‘lmerTest’ (Kuznetsova et al. 2017), ‘emmeans’ (Lenth 2020), ‘MuMIn’ (Bartón 2016) and ‘vegan’ (Oksanen et al. 2019).

## Results

### Plant biomass and litter quality

Aboveground vascular plant biomass (including litter) was highest at *B. nana* sites, followed by *E. hermaphroditum* and *A. alpinus* (Table 2). Root biomass exceeded aboveground vascular plant biomass at all sites except *E. hermaphroditum*. The deciduous shrubs exhibited markedly higher coarse root density compared to the other species, while only a marginally significant effect of plant species was observed for fine root density (F_5,20_ = 2.26, *p* = 0.09). Judged from visual inspection, roots in soil under mosses were mostly shrub roots intermixed with graminoid roots (M. Koranda, pers. obs.). Mosses exhibited strong interspecific differences in the mass of green and brown moss, which was threefold higher in *T. nitens* compared to *H. splendens*, exceeding total biomass and litter mass of the tallest shrub, *B. nana*.

**Table 2:** Plant biomass, leaf litter characteristics, soil characteristics and abiotic site factors at sites grown by the dwarf shrub species *Empetrum hermaphroditum, Arctostaphylos alpinus and Betula nana* and the moss species* Hylocomium splendens, Aulacomnium turgidum and Tomentypnum nitens*

	*E. hermaph*		*A. alpinus*		*B. nana*		*H. splendens*		*A. turgidum*		*T. nitens*	
*Plant biomass (including litter)*												
Aboveground vascular plants (kg DW m^−2^)	0.81 ^b^	(0.09)	0.44 ^c^	(0.03)	1.08 ^a^	(0.08)	0.16 ^d^	(0.05)	0.26 ^cd^	(0.03)	0.21 ^cd^	(0.01)
Moss (kg DW m^−2^)	0.02 ^c^	(0.02)	0.05 ^c^	(0.05)	0.10 ^c^	(0.08)	1.00 ^b^	(0.11)	2.24 ^a^	(0.12)	3.29 ^a^	(0.39)
Roots / rhizomes (kg DW m^−2^)	0.71 ^ab^	(0.06)	1.15 ^a^	(0.14)	1.38 ^a^	(0.26)	0.77 ^ab^	(0.11)	0.70 ^ab^	(0.08)	0.51 ^b^	(0.12)
Coarse root density (g DW dm^−3^)	5.4 ^ab^	(0.5)	9.2 ^a^	(2.1)	9.3 ^a^	(1.2)	4.7 ^ab^	(0.8)	4.6 ^ab^	(0.3)	3.4 ^b^	(0.9)
Fine root density (g DW dm^−3^)	3.4	(0.6)	3.2	(0.7)	3.7	(1.0)	2.8	(0.7)	2.9	(0.5)	2.0	(0.4)
*Leaf litter characteristics*												
Litter C (%)	59.5 ^a^	(0.3)	53.6 ^b^	(1.0)	52.9 ^b^	(0.1)	48.7 ^c^	(0.2)	47.4 ^c^	(0.2)	47.2 ^c^	(0.5)
Litter N (%)	0.93 ^a^	(0.00)	0.71 ^b^	(0.00)	0.68 ^b^	(0.02)	0.76 ^b^	(0.02)	0.99 ^a^	(0.04)	0.70 ^b^	(0.03)
Litter C:N ratio	64 ^c^	(1)	76 ^ab^	(1)	78 ^a^	(2)	64 ^c^	(2)	48 ^d^	(2)	68 ^bc^	(2)
Litter NIR spectra												
(PC 2 scores)	26.9 ^a^	(0.2)	17.2 ^b^	(0.3)	14.8 ^c^	(0.2)	-19.3 ^e^	(0.4)	-17.8 ^d^	(0.4)	-21.7 ^f^	(0.3)
(PC 3 scores)	1.9 ^c^	(0.4)	-14.9 ^e^	(0.1)	14.1 ^a^	(0.3)	-3.4 ^d^	(0.5)	-5.4 ^d^	(0.5)	7.7 ^b^	(1.2)
Condensed tannins (mg g^−1^ DW)	33 ^b^	(2)	34 ^b^	(1)	181 ^a^	(19)	n.d	n.d	0.3 ^c^	(0.1)	1.5 ^c^	(0.5)
*Soil characteristics*												
Soil C (%)	49.1 ^a^	(0.3)	48.6 ^a^	(0.6)	45.7 ^ab^	(1.8)	44.3 ^ab^	(1.8)	43.5 ^ab^	(1.4)	40.8 ^b^	(3.2)
Soil N (%)	1.23 ^b^	(0.01)	1.31 ^ab^	(0.10)	1.29 ^ab^	(0.03)	1.46 ^ab^	(0.06)	1.51 ^a^	(0.08)	1.38 ^ab^	(0.09)
Soil C:N ratio	40 ^a^	(0)	38 ^a^	(3)	36 ^ab^	(2)	31 ^b^	(1)	29 ^b^	(2)	30 ^b^	(3)
Soil NIR spectra (PC 2 scores)	1.00 ^a^	(0.32)	0.47 ^ab^	(0.41)	0.28 ^ab^	(0.25)	-0.78 ^c^	(0.19)	-0.81 ^c^	(0.07)	-0.18^bc^	(0.22)
Condensed tannins (mg g^−1^ DW)	4.6	(0.4)	5.1	(0.5)	7.0	(0.6)	6.2	(1.2)	4.3	(0.9)	4.2	(0.5)
Bulk density (g DW cm^−3^)	0.105 ^a^	(0.01)	0.107 ^a^	(0.01)	0.092 ^ab^	(0.01)	0.084 ^ab^	(0.01)	0.061 ^b^	(0.01)	0.066 ^b^	(0.01)
Organic horizon depth (cm)	8.1	(0.3)	9.5	(0.8)	10.3	(0.7)	10.2	(0.5)	9.3	(0.49	9.8	(0.6)
*Abiotic site factors*												
Average afternoon soil temperature (°C)	11.8 ^ab^	(0.6)	12.6 ^a^	(0.4)	10.7 ^b^	(0.1)	10.8 ^b^	(0.3)	12.0 ^ab^	0.4	11.1 ^ab^	(0.4)
Soil moisture (% of FW)	76.0 ^ab^	(1.0)	75.4 ^b^	(1.0)	74.6 ^b^	(1.0)	76.0 ^ab^	(1.0)	79.0 ^a^	0.5	77.6 ^ab^	(1.6)
Soil pH-value	5.1 ^b^	(0.1)	5.3 ^b^	(0.3)	5.7 ^b^	(0.2)	6.4 ^a^	(0.2)	6.9 ^a^	0.2	7.0 ^a^	(0.0)

Values are means (SE in parentheses), *n* = 5 (soil and site factors, root biomass), *n* = 4 (aboveground plant biomass), *n* = 3 (litter characteristics). Groups not sharing the same letter are significantly different (*p* < 0.05, Tukey’s post-hoc test). Soil characteristics, soil moisture and pH-values are mean values of two soil samplings (Data of separate samplings are given in Supplemental Information, Table S2), soil temperature data are means of four time points. NIR: Scores of the principal component analysis of Fourier transform near-infrared spectra. “n.d.”: not detected

We observed highly significant differences in leaf litter C content among plant species (F_5,12_ = 23.89, *p* < 0.001), which mirrored the gradient in C content of green leaves (Table S1). These contrasts likely reflect differences in content of lignin, which is generally well-correlated with C content in plant biomass and litter (Dorrepaal et al. 2005; Ma et al. 2018). There was no clear differentiation of shrubs and mosses in litter N content or C:N ratio. The relatively high leaf litter N content of *E. hermaphroditum* points to negligible resorption of N from senescent leaves in evergreen shrubs, in contrast to deciduous shrub species *A. alpinus* and *B. nana*, where N content in leaf litter was significantly lower (40% and 62%, respectively) than in green leaves. Analysis of FT-NIR spectra of leaf litter showed a clear separation of moss and shrub litter along the PC axis 2 (Table 2), caused by positive loadings of wavelengths characteristic of polysaccharides in shrub litter and negative loadings of wavelengths associated with aliphatic hydrocarbons in moss litter (Fig. S1). Separation along PC axis 3 was mainly related to resonance wavelengths of carbohydrates in *B. nana* and aromatic substances in *A. alpinus*. Leaf litter of *B. nana* was characterized by very high concentrations of condensed tannins (c. 18% of dry weight, Table 2), which was six-fold higher than in ericaceous shrub litter (*E. hermaphroditum* and *A. alpinus*), while in moss litter, condensed tannins were negligible.

### Soil characteristics and abiotic site factors

We observed a clear gradient in C content and C:N ratio of organic soil from ericaceous shrubs to mosses (plant species effect: F_5,20_ = 4.45, *p* < 0.01 and F_5,20_ = 6.97, *p* < 0.001, respectively, Table 2). This was mirrored by the FT-NIR spectra: Soil under *E. hermaphroditum* had highest scores on PC axis 2 positively associated with aliphatic hydrocarbons (Fig. S2), while soils under mosses *A. turgidum* and *H. splendens* were associated with resonance wavelengths of aromatic amines. Interestingly, scores of the moss *T. nitens* were intermediate. Concentrations of condensed tannins in soil showed only a marginally significant effect of plant species (F_5,20_ = 2.72, *p* = 0.05), in contrast to the strong differences observed in litter, but were also highest under *B. nana*.

There were no clear contrasts between moss and shrub sites in soil temperature, while soil moisture was highest under the mosses *A. turgidum* and *T. nitens*. We observed a strong gradient in soil pH-value (plant species effect: F_5,20_ = 27.12, *p* < 0.001), ranging from close to neutral pH at moss sites to acidic pH under ericaceous shrubs.

### Microbial community composition

**Fig. 3:**
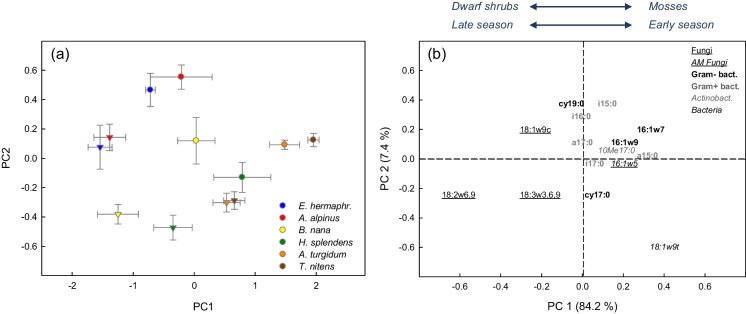
Sample scores (**a**) and variable loadings (**b**) of principal component analysis (PCA) of relative abundances of phospholipid fatty acids (PLFAs) in soil under the dwarf shrub species *Empetrum hermaphroditum, Arctostaphylos alpinus and Betula nana* and the moss species *Hylocomium splendens, Aulacomnium turgidum and Tomentypnum nitens* collected in early growing season (circles) and late growing season (triangles). Error bars indicate 1 SE. *n* = 5. Marker PLFAs for microbial groups are indicated by different font types. Three PLFAs with loadings close to zero are not depicted for reasons of clarity

Soil microbial community composition estimated from relative abundances of PLFAs clearly differed among plant species, plant functional types and season (Fig. 3a; PERMANOVA: plant species effect: F_5,48_ = 17.74, R^2^ = 0.51, *p* = 0.001; season: F_1,48_ = 35.50, R^2^ = 0.20, *p* = 0.001). Both the variation among plant species and seasons, mainly represented by the first PCA axis, was linked to differences in the fungi-to-bacteria ratio, i.e., shrub soils were characterized by fungal marker PLFAs and moss soils by bacterial marker PLFAs (Fig. 3b), and late season was characterized by fungal markers and early season by bacterial markers. The analysis of individual marker PLFAs showed that moss soils exhibited relatively high abundance of the bacterial PLFA 18:1ω9t, while the ericaceous shrub soils had high concentrations of Gram- marker cy19:0. Furthermore, early season soils were characterized by the Gram + marker i15:0, while late season soils, especially under *B. nana*, were characterized by the fungal marker 18:2ω6,9. It should be noted, however, that both the variation in microbial composition among plant species and seasonal changes between early and late growing season were mainly caused by strong differences in absolute abundance of fungal PLFAs (Tables 3 and 5), while only slight and not significant differences among plant species were found in total abundance of bacterial markers (plant species effect: *p* = 0.12).

**Table 3:** Soil carbon and nutrient availability, microbial biomass and abundance of marker PLFAs at sites grown by the dwarf shrub species *Empetrum hermaphroditum, Arctostaphylos alpinus and Betula nana* and the moss species *Hylocomium splendens, Aulacomnium turgidum and Tomentypnum nitens* in early and late growing season

		*E. hermaph*		*A. alpinus*		*B. nana*		*H. splendens*		*A. turgidum*		*T. nitens*	
*Dissolved org. C and nutrients*													
DOC (mg g^−1^ DW)	early	0.90 ^AB^	(0.07)	0.81 ^B^	(0.02)	0.97 ^A^	(0.08)	0.90 ^AB^	(0.07)	1.00 ^AB^	(0.12)	0.94 ^AB^	(0.16)
	late	0.74	(0.06)	0.73	(0.06)	1.21	(0.19)	0.94	(0.09)	0.98	(0.11)	0.88	(0.10)
DON (µg g^−1^ DW)	early	22 ^B^	(4)	18 ^B^	(6)	34 ^AB^	(6)	44 ^A^	(5)	58 ^A^	(6)	54 ^A^	(5)
	late	19	(9)	19	(2)	30	(8)	28	(6)	43	(8)	36	(7) >
DIN (µg g^−1^ DW)	early	9.9 ^ab^	(2.8)	5.5 ^b^	(0.9)	4.3 ^b^	(0.4)	10.4 ^ab^	(3.9)	16.3 ^a^	(4.5)	15.9 ^a^	(2.6)
	late	1.9 ^b^	(0.2)	3.7 ^ab^	(1.3)	3.0 ^ab^	(0.2)	4.1 ^ab^	(1.4)	7.8 ^a^	(1.2)	5.4 ^ab^	(1.9) >
PO_4_^−^ (µg g^−1^ DW)	early	1.5 ^B^	(0.2)	2.6 ^AB^	(1.4)	1.7 ^AB^	(0.5)	2.2 ^AB^	(0.3)	3.1 ^A^	(0.8)	2.0 ^AB^	(0.3)
	late	1.3	(0.3)	1.9	(0.3)	2.4	(0.3)	2.3	(0.4)	2.2	(0.3)	2.2	(0.3)
*Microbial biomass and community composition*													
Microbial biomass C (mg g^−1^ DW)	early	7.4	(0.2)	6.2	(0.0)	6.4	(0.8)	6.8	(0.2)	7.2	(0.6)	6.2	(0.3)
	late	8.7	(0.7)	7.5	(0.5)	8.9	(0.9)	8.3	(0.6)	7.7	(0.3)	7.5	(0.8) >
Microbial biomass N (mg g^−1^ DW)	early	0.62 ^B^	(0.03)	0.60 ^B^	(0.05)	0.66 ^B^	(0.06)	0.81 ^AB^	(0.06)	0.99 ^A^	(0.04)	1.00 ^A^	(0.13)
	late	0.61	(0.07)	0.55	(0.05)	0.75	(0.08)	0.83	(0.07)	1.02	(0.05)	1.00	(0.10)
Microbial biomass P (mg g^−1^ DW)	early	0.54 ^AB^	(0.05)	0.61 ^A^	(0.05)	0.46 ^AB^	(0.07)	0.46 ^AB^	(0.06)	0.48 ^AB^	(0.08)	0.36 ^B^	(0.04)
	late	0.46	(0.04)	0.54	(0.09)	0.48	(0.09)	0.40	(0.05)	0.41	(0.07)	0.28	(0.05) >
Microbial C:N ratio	early	14 ^A^	(1)	12 ^AB^	(1)	11 ^AB^	(1)	10 ^BC^	(1)	8 ^C^	(1)	8 ^C^	(1)
	late	17	(1)	16	(1)	14	(2)	12	(1)	9	(0)	9	(1) >
Microbial C:P ratio	early	37 ^AB^	(4)	27 ^B^	(2)	38 ^AB^	(5)	40 ^AB^	(5)	42 ^AB^	(6)	45 ^A^	(4)
	late	49	(3)	38	(4)	53	(7)	56	(5)	58	(15)	75	(11) >
Microbial N:P ratio	early	2.7 ^BC^	(0.3)	2.2 ^C^	(0.2)	3.4 ^BC^	(0.5)	4.2 ^AB^	(0.7)	5.1 ^AB^	(1.0)	6.1 ^A^	(0.7)
	late	2.9	(0.2)	2.4	(0.3)	4.0	(0.8)	4.9	(0.5)	6.9	(2.2)	8.7	(1.4) >
Bacterial marker PLFAs (nmol g^−1^ DW)	early	256	(9)	241	(23)	224	(28)	254	(12)	283	(9)	285	(23)
	late	238	(9)	229	(30)	284	(34)	248	(16)	303	(23)	362	(83)
Fungal marker PLFAs (nmol g^−1^ DW)	early	163 ^A^	(5)	125 ^AB^	(24)	108 ^AB^	(22)	98 ^ABC^	(22)	73 ^BC^	(16)	53 ^C^	(2)
	late	219	(22)	194	(38)	265	(59)	153	(23)	122	(15)	139	(35) >
Fungal: bacterial PLFAs	early	0.64 ^A^	(0.04)	0.53 ^AB^	(0.10)	0.49 ^AB^	(0.07)	0.39 ^BC^	(0.09)	0.26 ^CD^	(0.05)	0.19 ^D^	(0.01)
	late	0.92	(0.08)	0.83	(0.07)	0.89	(0.13)	0.62	(0.09)	0.41	(0.05)	0.38	(0.04) >

Values are means (SE in parentheses), *n* = 5. Uppercase letters indicate significant differences between plant species after 2-way ANOVA and Tukey’s post-hoc test, groups not sharing the same letter are significantly different (*p* < 0.05). Significant seasonal differences (*p* < 0.05) are indicated by “ > ” at the right end of the rows. Lowercase letters indicate differences between plant species after post-hoc tests run for early and late season in case of significant plant species x season interaction. Details on ANOVA models are presented in Table 5. “n.a” not analysed

### Soil carbon and nutrient pools and extracellular enzyme activities

**Fig. 4:**
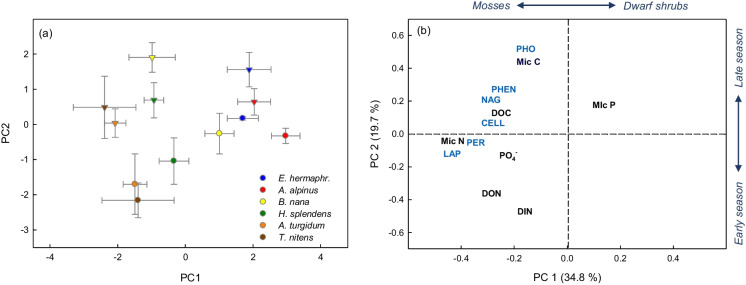
Sample scores (**a**) and variable loadings (**b**) of principal component analysis (PCA) of dissolved and microbial biomass carbon and nutrient pools and extracellular enzyme activities in soil under the dwarf shrub species *Empetrum hermaphroditum, Arctostaphylos alpinus and Betula nana* and the moss species *Hylocomium splendens, Aulacomnium turgidum and Tomentypnum nitens* collected in early growing season (circles) and late growing season (triangles). Error bars indicate 1 SE. *n* = 5. Enzyme activities are indicated in blue font: phosphatase (PHO), phenoloxidase (PHEN), N-acetyl-glucosaminidase (‘chitinase’, NAG), cellobiosidase (CELL), peroxidase (PER), leucine-aminopeptidase (LAP). Carbon and nutrient pools are indicated in black font: microbial biomass carbon (Mic C), dissolved organic carbon (DOC), dissolved organic nitrogen (DON), dissolved inorganic nitrogen (DIN)

As revealed by principal component analysis, soils taken under different plant species in early and late growing season, respectively, exhibited distinct patterns in availability of dissolved C and nutrients, microbial biomass C and nutrients and potential extracellular enzyme activities (Fig. 4a; PERMANOVA: plant species effect: F_5,48_ = 5.12, R^2^ = 0.30, *p* = 0.001; season: F_1,48_ = 8.52, R^2^ = 0.10, *p* = 0.001). Moss soils were characterized by high microbial biomass N and dissolved organic N (DON) concentrations, as well as high peptidase and peroxidase activities, while shrub soils were associated with high microbial biomass C and P and phosphatase activity (Fig. 4b, Tables 3 and 4). Soils collected in early growing season exhibited significantly higher dissolved N availability (DIN and DON) compared to late growing season, while the latter soils, especially under *B.nana*, were characterized by high levels of microbial biomass C, phosphatase, phenoloxidase and chitinase activities. The contrasts in microbial biomass C and nutrients between plant functional types and seasons resulted in strong gradients in microbial biomass stoichiometry: While microbial biomass in soil under the ericaceous shrub *E. hermaphroditum* exhibited on average 80% higher C:N ratio compared to the mosses *A. turgidum* and *T. nitens*, microbial N:P ratio under *A. alpinus* was 65% lower than under the mosses (Table 3). Furthermore, we observed a highly significant increase in microbial biomass C:N, C:P and N:P from early to late growing season at all sites (Tables 3 and 5). As shown by linear models, the observed strong contrasts in N pools and enzyme activities among plant species (Table 5) were also significant for plant functional type (moss versus shrub), with plant species included as random factor in the models (Table S3), which demonstrates that the grouping into the plant functional types mosses and shrubs is ecologically meaningful. Interestingly, the observed differences in C and nutrient pools and enzyme activities between mosses and shrubs changed, if values were calculated per soil volume instead of soil mass, due to 40% lower bulk soil density under mosses (*A. turgidum* and *T. nitens*) compared to ericaceous shrubs (Table 2): While differences in N pools and in enzyme activities associated with high N availability (peptidase and peroxidase) were markedly reduced or no longer significant per soil volume, shrub soils then exhibited significantly higher microbial biomass C and DOC compared to mosses, as well as significantly higher enzyme activities linked with high C availability, i.e. phosphatase, phenoloxidase and chitinase activity (Table S4).

**Table 4:** Extracellular enzyme activities in soil collected under the dwarf shrub species *Empetrum hermaphroditum, Arctostaphylos alpinus and Betula nana* and the moss species *Hylocomium splendens, Aulacomnium turgidum and Tomentypnum nitens* in early and late growing season

		*E. hermaph*		*A. alpinus*		*B. nana*		*H. splendens*		*A. turgidum*		*T. nitens*	
β-glucosidase (µmol MUF g^−1^ DW h^−1^)	early	0.88	(0.14)	0.95	(0.08)	0.91	(0.13)	1.02	(0.10)	1.24	(0.15)	1.24	(0.23)
	late	n.a		n.a		n.a		n.a		n.a		n.a	
Cellobiosidase (µmol MUF g^−1^ DW h^−1^)	early	0.23 ^AB^	(0.07)	0.13 ^B^	(0.02)	0.22 ^AB^	(0.09)	0.20 ^AB^	(0.03)	0.30 ^A^	(0.09)	0.27 ^A^	(0.05)
	late	0.11	(0.03)	0.12	(0.03)	0.27	(0.07)	0.24	(0.04)	0.35	(0.04)	0.35	(0.05)
Chitinase (µmol MUF g^−1^ DW h^−1^)	early	0.91	(0.06)	0.81	(0.07)	1.13	(0.11)	1.20	(0.18)	1.16	(0.12)	1.02	(0.17)
	late	0.88	(0.03)	0.99	(0.05)	1.45	(0.15)	1.20	(0.11)	1.24	(0.10)	1.26	(0.19) >
Peptidase (µmol AMC g^−1^ DW h^−1^)	early	0.08 ^C^	(0.01)	0.07 ^C^	(0.02)	0.12 ^BC^	(0.02)	0.15 ^AB^	(0.01)	0.21 ^AB^	(0.02)	0.22 ^A^	(0.03)
	late	0.09	(0.01)	0.09	(0.02)	0.13	(0.02)	0.16	(0.02)	0.22	(0.01)	0.24	(0.04) >
Phosphatase (µmol MUF g^−1^ DW h^−1^)	early	7.2	(0.3)	5.6	(0.5)	6.6	(0.6)	6.4	(0.8)	5.7	(0.7)	5.7	(0.7)
	late	7.9	(0.6)	6.9	(0.3)	8.7	(0.3)	6.9	(0.6)	7.5	(0.5)	8.7	(1.2) >
Phenoloxidase (µmol g^−1^ DW h^−1^)	early	2.6	(0.5)	2.5	(0.2)	2.4	(0.2)	2.8	(0.2)	2.7	(0.3)	3.0	(0.4)
	late	3.3	(0.4)	2.9	(0.3)	3.6	(0.3)	3.6	(0.3)	3.5	(0.3)	4.0	(0.4) >
Peroxidase (µmol g^−1^ DW h^−1^)	early	2.1 ^BC^	(0.5)	1.6 ^C^	(0.7)	2.9 ^BC^	(0.8)	4.4 ^AB^	(0.3)	4.2 ^AB^	(0.6)	6.1 ^A^	(0.7)
	late	3.2	(0.7)	2.7	(0.8)	4.3	(0.6)	6.5	(0.6)	5.9	(1.5)	8.0	(1.2) >

Values are means (SE in parentheses), *n* = 5. Uppercase letters indicate significant differences between plant species after 2-way ANOVA and Tukey’s post-hoc test, groups not sharing the same letter are significantly different (*p* < 0.05). Significant seasonal differences (*p* < 0.05) are indicated by “ > ” at the right end of the rows. Details on ANOVA models are presented in Table 5. “n.a” not analysed

**Table 5:** Summary of mixed-effect model ANOVA describing effects of plant species and seasonality on soil C and nutrient availability, microbial biomass and community composition and extracellular enzyme activities

	Plant species (df = 5)	Season (df = 1)	Species x season (df = 5)	R^2^_m_	R^2^_c_
Dissolved org. C and nutrients					
DOC ^a^	2.64 ^+^	0.06	1.09	0.22	0.40
DON ^a^	9.91 ***	8.48 **	0.94	0.49	0.65
DIN ^b^	4.32 **	65.46 ***	2.79 *	0.57	0.76
PO_4_^−^	2.72 *	0.20	1.14	0.25	0.25
Microbial biomass and community composition					
Microbial biomass C	1.76	33.29 ***	1.10	0.32	0.63
Microbial biomass N	10.05 ***	0.66	1.27	0.54	0.92
Microbial biomass P	2.60 ^+^	9.90 **	0.71	0.26	0.82
Microbial C:N ratio ^b^	14.04 ***	41.20 ***	1.26	0.67	0.89
Microbial C:P ratio ^b^	3.11 *	115.89 ***	0.85	0.46	0.88
Microbial N:P ratio ^b^	11.65 ***	15.96 ***	0.78	0.60	0.90
Bacterial PLFAs ^a^	2.00	1.11	0.99	0.22	0.40
Fungal PLFAs ^b^	6.10 **	55.47 ***	1.38	0.54	0.74
Fungi-to-bacteria ratio ^a^	18.34 ***	137.11 ***	0.85	0.69	0.91
Enzyme activities					
ß-glucosidase ^a^	1.29	-	-	0.16	0.28
Cellobiosidase ^a^	4.81 **	0.02	1.30	0.33	0.37
Chitinase	3.36 *	8.37 **	1.56	0.30	0.72
Peptidase ^b^	12.60 ***	19.05 ***	1.17	0.65	0.95
Phosphatase	1.71	58.07 ***	3.33 *	0.36	0.79
Phenoloxidase	1.12	26.05 ***	0.59	0.32	0.51
Peroxidase ^a^	7.03 ***	42.69 ***	0.12	0.55	0.89

Given are F-values for main effects and interaction. Significance levels: *** (*p* < 0.001), ** (*p* < 0.01), * (*p* < 0.05) and + (*p* < 0.1). Explained variance by fixed effects (R^2^_m_) and including random effects (R^2^_c_). ^a^ Square-root transformed data. ^b^ Log-transformed data

Using Mantel-tests, we found that the pattern of soil C and nutrient availability and enzyme activities under moss and shrub species was significantly related with the microbial community composition estimated from PLFA profiles (Mantel-statistic r: 0.44, *p* = 0.001). This relationship was also apparent if only enzyme activities were linked with PLFA profiles, excluding C and nutrient pools (Mantel-statistic r: 0.26, *p* = 0.001).

### Plant traits, soil characteristics and abiotic site factors explaining differences in microbial community composition under plant functional types

**Fig. 5:**
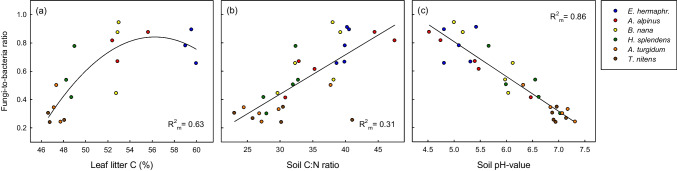
Relationships of fungi-to-bacteria ratio (estimated from PLFAs) with (**a**) leaf litter C (**b**) soil C:N ratio and (**c**) soil pH-value at sites grown by the dwarf shrub species *Empetrum hermaphroditum, Arctostaphylos alpinus and Betula nana* and the moss species *Hylocomium splendens, Aulacomnium turgidum and Tomentypnum nitens*. Values are growing season averages, *n* = 30 (except for (**a**), where *n* = 18). Regression lines are given to depict the shape of relationships. Note that figures show original untransformed data, while for regression models fungi-to-bacteria ratio and soil C:N ratio were square-root transformed. Marginal R.^2^ of simple mixed model regressions is indicated in the figures. For details on multiple regression models and significance of predictors see Table 6

Regression analysis revealed that the variation in fungi-to-bacteria ratio under different plant species was best explained by the model including only the site factor soil pH-value as predictor (Table 6 and Fig. 5c), followed by the plant trait model including root morphological traits (coarse root and fine root density) and litter C content (Fig. 5a) as predictors. Soil C:N ratio (Fig. 5b) and soil NIR spectra also significantly explained the fungi-to-bacteria ratio, but the variance explained by soil characteristics as fixed predictors was lower compared to the plant trait and site factor models. Results of the regression models with the PC 1 scores of the PLFA ordination as dependent variable were very similar to the models using fungi-to-bacteria ratio as dependent variable, with slightly higher explained variance (Table S5), which shows that overall differences in microbial community composition at this tundra site were well characterized by the fungi-to-bacteria ratio.

**Table 6:** Summary of best linear mixed-effect regression models describing the relationship of fungi-to-bacteria ratio (estimated from PLFAs) with selected plant traits, soil characteristics and abiotic site factors as explanatory variables

Plant factors	*t*-value	Soil factors	*t*-value	Site factors	*t*-value
(Intercept)	-4.95 **	(Intercept)	0.63	(Intercept)	19.94 ***
Coarse root density	3.58 **	Soil C:N ratio	2.07 *	Soil pH	-11.85 ***
Fine root density	-2.55 *	Soil NIR PC2	1.9 ^+^		
Leaf litter % C	5.48 **				
R^2^_m_ / R^2^_c_	**0.71** / 0.74	R^2^_m_ / R^2^_c_	**0.42** / 0.71	R^2^_m_ / R^2^_c_	**0.86** / 0.88

Significance levels: *** (*p* < 0.001), ** (*p* < 0.01), * (*p* < 0.05) and + (*p* < 0.1). Explained variance by fixed predictors (R^2^_m_ in bold), and including plant species random effect (R^2^_c_). Regressions were run with growing season averages. *n* = 30. Data were square-root transformed (fungi-to-bacteria ratio, soil C:N ratio) or log-transformed (root density) to achieve normal distribution

## Discussion

In this study we investigated divergent effects of two dominant plant functional types in tundra heath, dwarf shrubs and mosses, on soil microbial communities and microbial processes, and elucidated the factors responsible for these effects on soil functioning. Although our study did not consider all parameters possibly relevant for plant-soil interactions in this ecosystem (e.g., we did not directly measure root exudation or plant nutrient uptake and snow depth in winter), our comprehensive data set sheds new light on the complex interactions between plant functional types and soil microbial communities and their impact on carbon and nutrient cycling in tundra ecosystems.

### Effects of mosses on microbial decomposition processes and soil nutrient availability

Contrary to our first hypothesis, soils at moss-grown sites exhibited relatively high microbial biomass N, dissolved N and potential enzyme activities (Fig. 4, Tables 3 and 4), which contrasts with findings of our previous study in a nearby birch forest, where we observed a negative effect of the moss layer on soil N availability (Koranda and Michelsen 2021). This apparent discrepancy shows that the influence of mosses on soil microbial processes and nutrient cycling is context-specific, depending on the factors most strongly regulating soil microbial activity in the respective ecosystem: While in the birch forest, characterized by a homogenous understorey and SOM quality, the insulating effect of the moss layer was a crucial factor for soil microbial activity (Koranda and Michelsen 2021), in tundra heath the high spatial variability in plant traits, SOM quality and soil pH mainly determined microbial community structure and nutrient availability, whereas soil temperature was comparatively less relevant.

Our results thus raise the following question: Why does moss litter decompose so slowly compared to vascular plant litter (Dorrepaal et al. 2005; Cornwell et al. 2008), although organic soil under mosses (consisting at a high proportion of decomposing moss litter) exhibits high microbial biomass and enzyme activities? One explanation for this apparent contradiction probably lies in the microbial community structure. As shown by PLFA profiles (Fig. 3), the microbial community at moss-grown sites was bacterial-dominated, while the main decomposers of plant litter are saprotrophic fungi (Rousk and Bååth 2011; Koranda et al. 2014). It is thus likely that the majority of bacterial taxa in moss soils do not thrive on decomposition of the moss litter, but rather on dissolved organic matter leached from the living moss or washed in via subsurface water flows, or on decomposition of SOM and recycling of bacterial necromass. This view is supported by a study from Alaskan tundra reporting Gram + bacteria as proteolytic specialists (McMahon and Schimel 2017), and by previous results from Abisko area showing that peptidase and peroxidase activities generally correlated with bacterial abundance in organic soils (Koranda and Michelsen 2021, and unpublished data). The reason for the close linkage of peptidase and peroxidase activities in moss soils (Fig. 4b) might be that oxidative enzymes are needed for liberation of insoluble SOM-bound proteins (Weintraub and Schimel 2005), which suggests that peptidase and peroxidase enzymes are produced by associated (or identical) bacterial taxa.

Various theories have been put forward to explain the low decomposition rates of moss litter, such as low litter N content (Hobbie 1996), high content of phenolics (Verhoeven and Toth 1995) or lignin-like compounds in moss litter (Bengtsson et al. 2018), which are, however, not supported by our data. Alternative explanations might be related to other secondary metabolites besides phenolics in moss biomass, such as terpenoids (Kanerva et al. 2008), or the high content of storage lipids in mosses (Koranda et al. 2007), which was also indicated by the NIR spectra of moss litter in our study (Fig. S1 and Table 2), and which possibly makes moss litter a less attractive substrate for (fungal) decomposers compared to vascular plant litter. It is worth mentioning that we observed significant differences among moss species in the depth of the layer of intact, brown moss (see moss biomass in Table 2), likely owing to differences in decomposition rates, which might partly be related to the contrasting morphology of the moss species (Table 1).

### Differences in shrub effects: Ericaceous dwarf shrubs versus ectomycorrhizal shrubs

Ericaceous dwarf shrubs and their mycorrhizal symbionts are known to promote a ‘closed’ N-cycle via recycling of their own litter (Read et al. 2004; Martino et al. 2018), thereby creating and maintaining nutrient-poor conditions in order to prevent competition by other plant species. This was supported by our data, showing that DON concentrations under ericaceous shrubs were on average 60% lower compared to moss soils (*A. turgidum* and *T. nitens*) and 40% lower than under *B. nana*, combined with low microbial biomass N and low peptidase and peroxidase activities (Tables 3 and 4). The negative effect of ericaceous shrubs on soil nutrient availability and microbial activity is often attributed to a high content of phenolic compounds, especially condensed tannins, which form stable complexes with organic N compounds and may reduce enzyme activities (Adamczyk et al. 2011, 2017). Concentrations of condensed tannins in leaf litter of ericaceous shrubs were, however, only one sixth compared to *B. nana* leaf litter. But ericaceous shrubs also contain significant amounts of soluble phenolics (Dorrepaal et al. 2007), which may have antimicrobial or allelophatic effects (Wardle et al. 1998; Fierer et al. 2001). While our results demonstrated a negative effect of ericaceous shrubs on soil N availability and enzyme activities, they revealed a positive influence on soil C content (Table 2). This likely not only reflects limitation of soil microbial activity by low N availability and/or toxic effects, but may also be linked to the recalcitrant nature of the melanized hyphae of ericoid mycorrhizal fungi (Clemmensen et al. 2015; Fernandez and Kennedy 2018), and to high concentrations of cuticular waxes in evergreen shrub litter (Parker et al. 2018).

*B. nana* soil, on the other hand, which was intermediate between ericaceous shrubs and mosses in many soil variables, exhibited the greatest seasonal variation in microbial biomass and extracellular enzyme activities, most likely owing to the high aboveground and belowground biomass of *B. nana*. Plant belowground C allocation over the growing season resulted in a strong increase in labile soil C pools under *B. nana* from early to late growing season (25% and 40% increase in DOC and microbial biomass C, respectively) as well as a 2.5-fold increase in abundance of fungal PLFAs (Table 3). The enhanced C availability also led to a strong stimulation of phosphatase, phenoloxidase and chitinase activities in late growing season (Table 4), indicating a priming effect of microbial decomposition processes by fresh plant C supply (Hicks et al. 2020; Keuper et al. 2020). It is, however, uncertain, whether this effect is attributable to (free-living) rhizosphere microbes or to mycorrhizal symbionts, as the ‘short-distance exploration type’ ectomycorrhizal fungal species associated with dwarf shrubs generally have lower capacity for enzyme production compared to EM fungi associated with trees (Clemmensen et al. 2021; but see also Dunleavy and Mack 2021). Interestingly, the high content of condensed tannins in *B. nana* leaf litter (Table 2) apparently did not impair soil microbial activity, possibly owing to relatively high degradability depending on the chemical structure of tannins (Nierop et al. 2006). The lack of strong differences among plant species in concentrations of tannins in soil suggests lateral translocation of degradation products (Hättenschwiler and Vitousek 2000), likely during water-logged conditions in spring.

### Plant functional types as drivers of microbial community composition

As shown by the discussion above, soil microbial community structure was closely linked with enzyme activities and soil C and nutrient availability (see also Mantel-coefficients) and is hence likely a crucial factor in the relationship of plant functional types with soil functions. An important question is thus which factors caused the strong contrasts in soil microbial community composition we observed under different plant species in this tundra heath (Fig. 3). As shown by the regression models, the variation in fungi-to-bacteria ratio, as a measure of microbial community structure, was best explained by soil pH (Table 6, Fig. 5c). While a similar linkage has previously been described in studies comparing different vegetation types (Högberg et al. 2006; Eskelinen et al. 2009; Gavazov et al. 2022), we found this relationship at one study site, where pH varied by more than two units within a few metres distance. Although it is well-known that bacterial and fungal growth differs in pH-optimum (Rousk and Bååth 2011), the relationship of soil pH with fungi-to-bacteria ratio at our study site was, however, likely rather of correlative than causal nature, i.e., linked to plant species: As reported by Nilsson et al. (2005), 57% of the fungal marker PLFAs in an acidic boreal forest were of ericoid/ectomycorrhizal origin, and the acidifying properties of ericaceous shrubs are well-known (Adamczyk et al. 2016). Additionally, input of DOM via base cation-rich subsurface water flow may have stimulated bacterial growth in the close-to-neutral moss soils. Regression models also showed that fungi-to-bacteria ratio was well explained by plant traits characterising the different plant species: root morphological traits, i.e. the ratio of coarse root and fine root biomass, and leaf litter C content (Table 6, Fig. 5), whereas measures of SOM quality were less significant predictors of microbial community structure than plant traits. The relatively broad range in soil C:N ratio and fungi-to-bacteria ratio under *B. nana* and *A. alpinus* (Fig. 5b) suggests that deciduous shrubs (but not the evergreen shrubs) regulate belowground C allocation to mycorrhizal symbionts depending on soil nutrient availability, as previously described for boreal forests (Högberg et al. 2007, 2010). Our results hence indicate that plant species and their mycorrhizal symbionts were the main drivers of soil microbial community structure at this tundra site. While the pronounced plant-driven spatial variation in microbial community composition was independent of seasonality, we found a general increase in fungal abundance from early to late growing season under all plant species (although most pronounced under *B. nana*) (Table 3), likely reflecting plant photosynthetic activity and increased availability of fresh C substrates over the summer. Our results thus revealed a stronger influence of plant traits on the soil fungal community than on the bacterial community during the growing season (at the coarse resolution of PLFAs), which is in line with studies from Alaskan shrub tundra using metagenomic sequencing data (Deslippe et al. 2012; Pold et al. 2021).

### Implications for ecosystem C and nutrient cycling

There were two main findings of our study concerning the functioning of tundra ecosystems: Firstly, our results demonstrated that moss-grown sites were ‘hotspots’ of soil N-availability and N-cycling, with potential for ecosystem N-losses, but also lateral N translocation, whereas shrubs promoted soil C storage. High soil C content and bulk density of shrub soils resulted in (on average) 50% higher soil C stocks per area compared to moss soils (4.4 versus 2.9 kg C m^−2^; Fig. S3). Even if living and dead plant biomass were included in the calculations, estimated total C stocks at shrub sites exceeded those at moss sites, despite the thick layers of undecomposed brown moss (5.4 versus 4.4 kg C m^−2^).

Secondly, we found that the seasonal variation in enzyme activities and microbial biomass over the summer was most pronounced under the tallest shrub *B. nana*, which implies that the ongoing expansion of deciduous shrubs in the Arctic will likely amplify temporal dynamics of microbial decomposition processes (and hence soil priming effects) over the growing season, an aspect, which has generally been overlooked in the discussion on the ‘Arctic greening’.

## Conclusion

Our study showed that plant functional types / plant species and their close coupling with soil microbial communities drive a high spatial and temporal variation in extracellular enzyme activities and soil C and nutrient availability in tundra heath. The spatial variation in microbial decomposition processes and microbial community structure was driven by plant traits like mycorrhizal association, root morphology and plant litter quality, but was also linked with SOM quality and the mainly indirect (i.e., plant species-related) effect of soil pH. The temporal variation in soil functioning and microbial biomass over the growing season was likely driven by plant photosynthetic activity (related with photosynthetic leaf area and plant biomass in general) and thus plant belowground C allocation during the summer.

The intimate linkage of plant functional types with soil microbial communities, microbial decomposition processes and soil nutrient availability demonstrated in our study hence suggests potential strong impacts of global change-induced shifts in plant community composition on soil nutrient availability and C storage in high-latitude ecosystems, which possibly surpass direct effects of climate change on ecosystem C and nutrient cycling.

## Supplementary Information

Below is the link to the electronic supplementary material.

Supplementary file1 (PDF 480 KB)

## Data Availability

The data that support the findings of this study are available at the ‘Mendeley Data’ repository (https://doi.org/10.17632/4gn7tk33ph.1).
