# Supplementary material for: Close coupling of plant functional types with soil microbial community composition drives soil carbon and nutrient cycling in tundra heath
Source: Plant Soil. 2023 Mar 27;488(1-2):551–72. doi: 10.1007/s11104-023-05993-w (PMC10435393; doi:10.1007/s11104-023-05993-w)
Supplement: Supplementary file 1 — Supplementary file1 (PDF 480 KB) [file 11104_2023_5993_MOESM1_ESM.pdf]

Supporting Information to the article 'Close coupling of plant functional types with soil microbial community composition drives soil carbon and nutrient cycling in tundra heath'

Plant and Soil (2023)

Marianne Koranda *, Riikka Rinnan and Anders Michelsen

* Corresponding author: M. Koranda, Division of Terrestrial Ecosystem Research, Centre for Microbiology and Environmental Systems Science, University of Vienna, 1030 Vienna, Austria. E-mail: marianne.koranda@univie.ac.at.

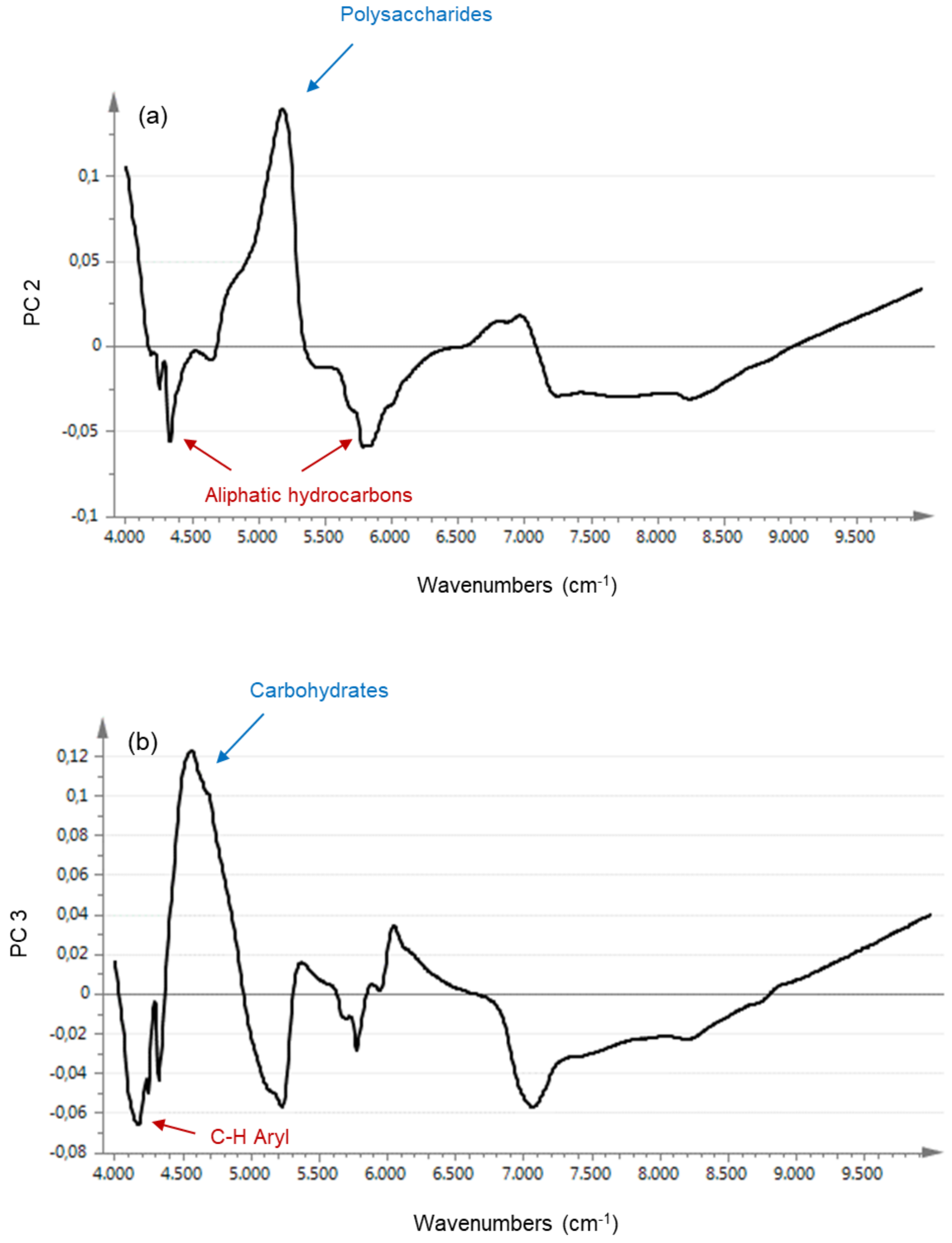

Fig. S1 Loadings of the second (a) and the third (b) axis of principal component analysis (PCA) of Fourier transform near-infrared (FT-NIR) spectra measured from leaf litter samples of three dwarf shrub and three moss species. Arrows indicate suggested assignment of wavenumbers to chemical bonding types and compound classes.

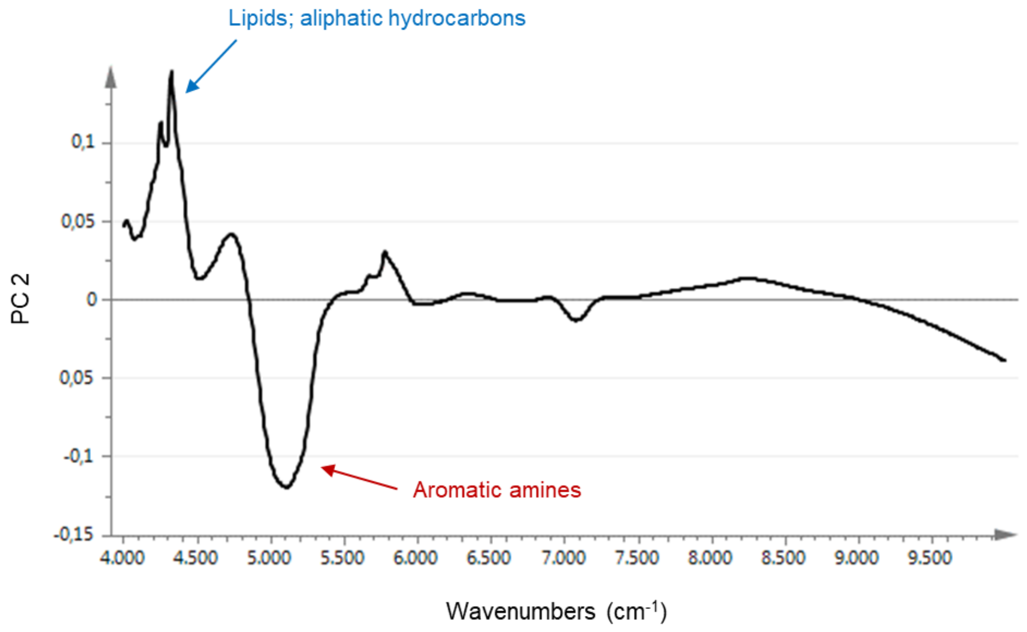

Fig. S2 Loadings of the second axis of principal component analysis (PCA) of Fourier transform near-infrared (FT-NIR) spectra measured from soil samples collected under three dwarf shrub and three moss species. Arrows indicate suggested assignment of wavenumbers to chemical compound classes.

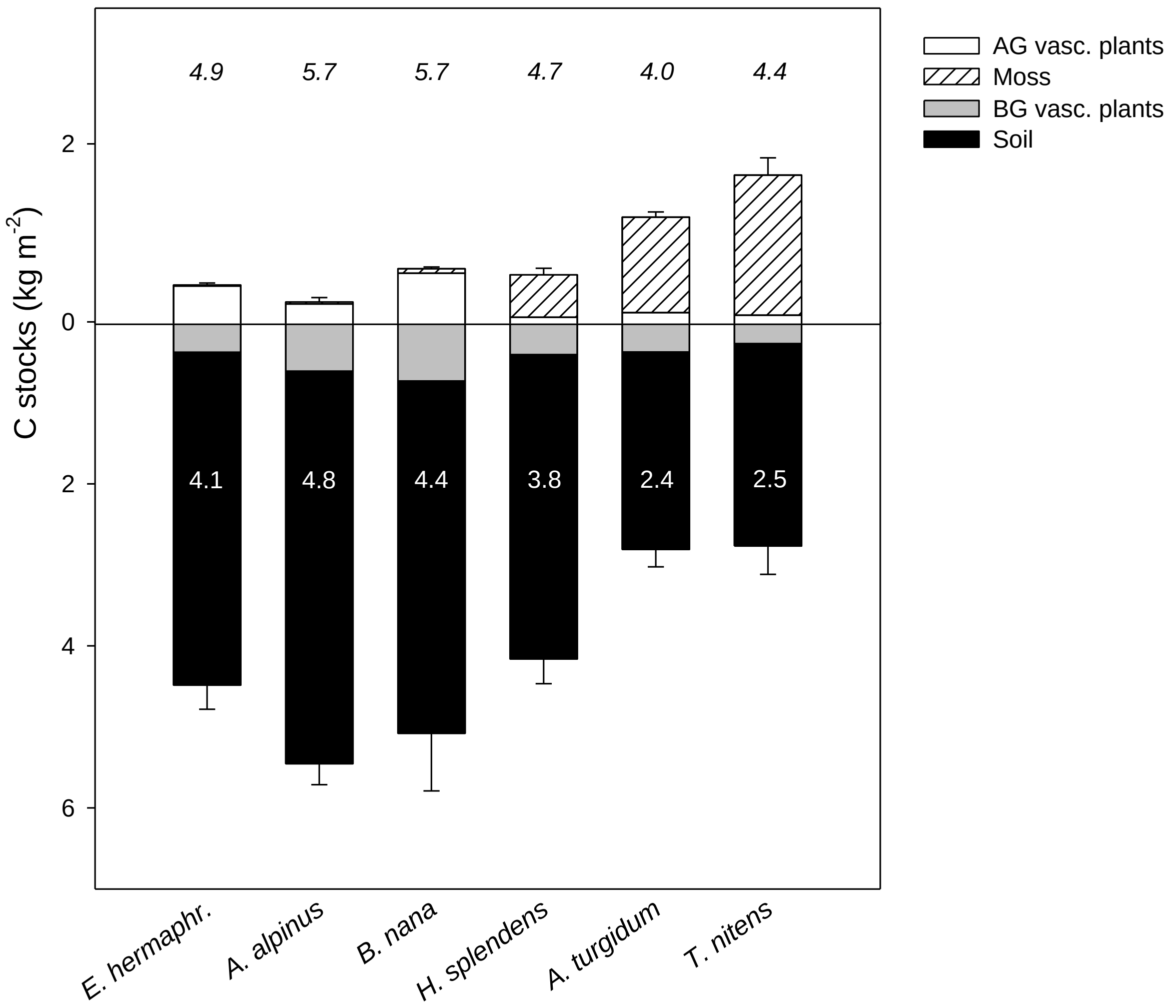

Fig. S3 Estimated C stocks in organic soil and plant biomass (including litter) at sites grown by the dwarf shrub species *Empetrum hermaphroditum, Arctostaphylos alpinus and Betula nana* and the moss species *Hylocomium splendens, Aulacomnium turgidum and Tomentypnum nitens.* C stocks in plant biomass are rough estimations assuming an overall C content of 53 % for vascular plant biomass and litter and 47 % for moss biomass. Numbers inside the black bars indicate soil C stocks. Numbers in italics on top indicate total C stocks including plant biomass. Values are means, n = 5. Error bars indicate 1 SE for above- and belowground C stocks, respectively.

.

Table S1 Characteristics of green leaves of the dwarf shrub species *Empetrum hermaphroditum, Arctostaphylos alpinus and Betula nana* and the moss species *Hylocomium splendens, Aulacomnium turgidum and Tomentypnum nitens*.

	E. hermaph.		*A. alpinus*		*B. nana*		*H. splendens*		*A. turgidum*		*T. nitens*	
Leaf C (%)	55.0 [a]	(0.2)	51.4 [bc]	(0.9)	53.8 [ab]	(0.5)	49.8 [cd]	(1.2)	47.2 [de]	(0.3)	46.4 [e]	(0.6)
Leaf N (%)	0.8 [bc]	(0.0)	1.2 [b]	(0.1)	1.8 [a]	(0.2)	0.5 [c]	(0.0)	0.7 [c]	(0.1)	0.7 [c]	(0.1)
Leaf C:N ratio	65 [b]	(3)	42 [bc]	(3)	31 [c]	(3)	100 [a]	(3)	73 [b]	(8)	70 [b]	(7)

Values are means (SE in parentheses), n = 3. Groups not sharing the same letter are significantly different ($p < 0.05$, Tukey's post-hoc test).

Table S2 Soil characteristics and abiotic site factors at sites grown by the dwarf shrub species *Empetrum hermaphroditum, Arctostaphylos alpinus and Betula nana* and the moss species *Hylocomium splendens, Aulacomnium turgidum and Tomentypnum nitens* in early and late growing season.

		E. hermaph.		*A. alpinus*		*B. nana*		*H. splendens*		*A. turgidum*		*T. nitens*	
**Soil characteristics**													
Soil % C	early	48.7	(0.6)	49.1	(0.4)	44.8	(1.6)	43.9	(2.2)	43.0	(1.3)	42.6	(3.5)
	late	49.5	(0.2)	48.0	(1.1)	46.6	(2.0)	44.8	(1.5)	44.1	(1.6)	39.0	(3.0)
Soil % N	early	1.22	(0.02)	1.32	(0.11)	1.29	(0.04)	1.51	(0.05)	1.46	(0.08)	1.37	(0.09)
	late	1.24	(0.02)	1.30	(0.10)	1.29	(0.03)	1.40	(0.06)	1.57	(0.08)	1.39	(0.11)
Soil C:N ratio	early	40	(1)	38	(3)	35	(2)	29	(1)	30	(2)	32	(3)
	late	40	(1)	38	(3)	36	(2)	32	(1)	28	(2)	29	(3)
NIR spectra	early	0.53	(0.34)	0.18	(0.56)	-0.10	(0.26)	-1.07	(0.27)	-1.21	(0.10)	-0.52	(0.35)
(PC 2 scores)	late	1.46	(0.33)	0.77	(0.30)	0.66	(0.26)	-0.48	(0.12)	-0.40	(0.15)	0.16	(0.19)
**Abiotic site factors**													
Soil temp. (°C)	early	7.8	(1.2)	8.6	(1.0)	6.7	(0.4)	6.5	(0.5)	7.0	(0.3)	7.0	(0.5)
	late	9.1	(0.3)	9.4	(0.2)	8.9	(0.1)	9.2	(0.2)	9.3	(0.2)	9.0	(0.2)
Soil moisture	early	75.2	(1.3)	74.6	(1.1)	73.1	(0.9)	75.2	(1.2)	78.6	(0.6)	77.4	(1.6)
(% of FW)	late	76.7	(0.7)	76.2	(1.0)	76.2	(1.1)	76.9	(0.9)	79.4	(0.3)	77.8	(1.8)
Soil pH-value	early	5.1	(0.2)	5.4	(0.3)	5.7	(0.2)	6.4	(0.2)	6.9	(0.2)	7.0	(0.1)
	late	5.1	(0.1)	5.2	(0.4)	5.6	(0.3)	6.3	(0.3)	7.0	(0.2)	6.9	(0.1)

Values are means (SE in parentheses), n = 5.

Table S3 Summary of mixed-effect model ANOVA describing effects of plant functional type (PFT, i.e. shrubs versus mosses), and seasonality on soil C and nutrient availability, soil microbial biomass and community composition and extracellular enzyme activities.

	PFT (df = 1)	Season (df = 1)	PFT x season (df = 1)	R^2_m	R^2_c
**Dissolved org. C and nutrients**					
DOC [a]	0.25	0.06	0.04	0.01	0.35
DON [a]	12.12 *	9.62 **	4.41 *	0.39	0.68
DIN [b]	16.03 *	49.29 ***	0.35	0.49	0.65
PO_4^-	6.83 [+]	0.15	1.46	0.15	0.17
**Microbial biomass and community composition**					
Microbial biomass C	0.36	33.10 ***	1.31	0.24	0.61
Microbial biomass N	18.46 *	0.61	0.05	0.45	0.91
Microbial biomass P	5.29 [+]	10.32 **	0.75	0.17	0.81
Microbial C:N ratio [b]	15.89 *	42.83 ***	3.49 [+]	0.55	0.89
Microbial C:P ratio [b]	4.31	116.78 ***	0.46	0.36	0.88
Microbial N:P ratio [b]	12.81 *	17.80 ***	3.14 [+]	0.46	0.91
Bacterial PLFAs [a]	4.89 [+]	1.12	0.19	0.11	0.31
Fungal PLFAs [b]	16.80 *	51.45 ***	0.43	0.48	0.70
Fungi-to-bacteria ratio [a]	17.84 *	144.23 ***	1.16	0.60	0.91
**Enzyme activities**					
ß-glucosidase [a]	5.48 *	-	-	0.13	0.29
Cellobiosidase [a]	6.48 [+]	0.02	2.60	0.20	0.33
Chitinase	1.31	7.45 *	0.27	0.10	0.68
Peptidase [b]	15.10 *	20.05 ***	3.42 [+]	0.52	0.95
Phosphatase	0.50	40.94 ***	0.67	0.23	0.69
Phenoloxidase	4.29 *	25.89 ***	0.19	0.30	0.47
Peroxidase [a]	21.06 *	48.93 ***	0.00	0.50	0.90

Given are F-values for main effects and interaction. Plant species is included as random effect in the models. Significance levels: *** ($p<0.001$), ** ($p<0.01$), * ($p<0.05$) and + ($p<0.1$). Explained variance by fixed effects (R^2_m) and including random effects (R^2_c). [a] Square-root transformed data. [b] Log-transformed data.

Table S4 Carbon and nutrient availability, microbial biomass and extracellular enzyme activities (values per soil volume) at sites grown by the dwarf shrub species *Empetrum hermaphroditum, Arctostaphylos alpinus and Betula nana* and the moss species *Hylocomium splendens, Aulacomnium turgidum and Tomentypnum nitens* in early and late growing season.

		E. hermaph.		*A. alpinus*		*B. nana*		*H. splendens*		*A. turgidum*		*T. nitens*	
**Dissolved org. C and nutrients**													
DOC (µg cm^{-3})	early	94 ^{A}	(11)	86 ^{A}	(6)	88 ^{A}	(7)	74 ^{AB}	(4)	59 ^{BC}	(5)	55 ^{C}	(3)
	late	76	(7)	77	(7)	113	(26)	79	(8)	59	(8)	55	(6)
DON (µg cm^{-3})	early	2.3	(0.5)	1.9	(0.6)	3.4	(0.9)	3.7	(0.5)	3.5	(0.5)	3.3	(0.4)
	late	1.9	(0.9)	2.0	(0.1)	3.0	(1.0)	2.4	(0.6)	2.7	(0.7)	2.4	(0.6) >
Inorganic N (µg cm^{-3})	early	0.98	(0.21)	0.58	(0.09)	0.39	(0.04)	1.15	(0.46)	1.04	(0.33)	0.99	(0.17)
	late	0.20	(0.02)	0.37	(0.10)	0.28	(0.04)	0.33	(0.10)	0.50	(0.11)	0.35	(0.13) >
PO_4^- (µg cm^{-3})	early	0.16	(0.01)	0.12	(0.04)	0.18	(0.07)	0.19	(0.03)	0.20	(0.05)	0.13	(0.02)
	late	0.13	(0.04)	0.21	(0.04)	0.21	(0.02)	0.20	(0.03)	0.14	(0.03)	0.13	(0.02)
**Microbial biomass**													
Microbial biomass C	early	773 ^{A}	(68)	663 ^{A}	(43)	591 ^{A}	(105)	572 ^{AB}	(58)	426 ^{BC}	(21)	397 ^{C}	(54)
(µg cm^{-3})	late	910	(129)	802	(66)	804	(105)	693	(56)	476	(71)	466	(58) >
Microbial biomass N	early	65	(6)	64	(6)	62	(10)	68	(7)	60	(5)	60	(5)
(µg cm^{-3})	late	64	(10)	58	(4)	70	(11)	69	(6)	62	(8)	61	(5)
Microbial biomass P	early	54 ^{AB}	(3)	65 ^{A}	(6)	40 ^{ABC}	(3)	38 ^{BC}	(4)	28 ^{CD}	(4)	23 ^{D}	(3)
(µg cm^{-3})	late	48	(7)	57	(10)	41	(6)	33	(4)	25	(6)	17	(3) >
**Enzyme activities**													
β-glucosidase	early	97	(20)	100	(6)	83	(13)	85	(7)	75	(12)	73	(8)
(nmol MUF cm^{-3} h^{-1})	late	n.a.		n.a.		n.a.		n.a.		n.a.		n.a.	
Cellobiosidase	early	22	(5)	14	(2)	18	(6)	16	(2)	17	(4)	17	(3)
(nmol MUF cm^{-3} h^{-1})	late	12	(4)	14	(4)	26	(9)	21	(5)	22	(5)	21	(2)

Chitinase	early	95 ^{AB}	(11)	85 ^{AB}	(6)	105 ^{A}	(15)	100 ^{AB}	(15)	70 ^{B}	(8)	63 ^{B}	(10)
(nmol MUF cm^{-3} h^{-1})	late	93	(12)	105	(8)	132	17	103	(17)	76	(11)	78	(13) >
Peptidase	early	8	(1)	8	(2)	11	(2)	12	(1)	13	(2)	13	(0)
(nmol AMC cm^{-3} h^{-1})	late	9	(1)	10	(2)	13	(3)	13	(2)	14	(2)	15	(1) >
Phosphatase	early	753 ^{A}	(79)	604 ^{A}	(61)	610 ^{A}	(86)	539 ^{AB}	(80)	346 ^{B}	(47)	347 ^{B}	(23)
(nmol MUF cm^{-3} h^{-1})	late	822	(93)	740	(51)	809	(97)	584	(74)	459	(57)	526	(40) >
Phenoloxidase	early	269 ^{A}	(58)	267 ^{AB}	(19)	217 ^{AB}	(19)	232 ^{AB}	(20)	169 ^{B}	(27)	182 ^{AB}	(22)
(nmol cm^{-3} h^{-1})	late	330	(16)	300	(17)	353	(46)	301	(15)	220	(40)	247	(23) >
Peroxidase	early	221	(73)	178	(78)	292	(99)	378	(64)	255	(38)	382	(48)
(nmol cm^{-3} h^{-1})	late	333	(81)	302	(88)	435	(101)	532	(39)	369	(95)	517	(95) >

Values are means (SE in parentheses), n = 5. Uppercase letters indicate significant differences between plant species after 2-way ANOVA and Tukey's post-hoc test, groups not sharing the same letter are significantly different ($p<0.05$). Significant seasonal differences ($p<0.05$) are indicated by ">" at the right end of the rows. "n.a" not analysed.

Table S5 Summary of best linear mixed-effect regression models describing the relationship of microbial community composition (estimated from PC1 scores of the ordination of PLFAs) with selected plant traits, soil characteristics and abiotic site factors as explanatory variables.

**Plant factors**	t-value	**Soil factors**	t-value	**Site factors**	t-value
(Intercept)	7.52 **	(Intercept)	1.94 $^+$	(Intercept)	-17.26 ***
Coarse root density	-4.29 ***	Soil C:N ratio	-1.96 $^+$	Soil pH	17.45 ***
Fine root density	2.76 *	Soil NIR PC2	-2.56 *		
Leaf litter % C	-6.39 **				
R^2_m / R^2_c	**0.77** / 0.79	R^2_m / R^2_c	**0.44** / 0.77	R^2_m / R^2_c	**0.93** / 0.93

Significance levels: *** (p<0.001), ** (p<0.01), * (p<0.05) and + (p<0.1). Explained variance by fixed predictors (R^2_m in bold), and including plant species random effect (R^2_c). Regressions were run with growing season averages. n = 30. Data were square-root transformed (soil C:N ratio) or log-transformed (root density) to achieve normal distribution.
